# Tumor budding as a predictor of disease-free survival in patients with cholangiocarcinoma

**DOI:** 10.3389/pore.2023.1611216

**Published:** 2023-05-18

**Authors:** Kyung Bin Kim, Ji Hyun Ahn, Soon Wook Kwon, Su Ji Lee, Yury Lee, Seo Young Park, Ahrong Kim, Kyung Un Choi, Chang Hun Lee, Gi Yeong Huh

**Affiliations:** ^1^ Department of Pathology, Pusan National University Hospital, Busan, Republic of Korea; ^2^ Department of Pathology, Pusan National University School of Medicine, Yangsan-si, Gyeongsangnamdo, Republic of Korea

**Keywords:** prognosis, cholangiocarcinoma, tumor budding, prediction, disease-free survival

## Abstract

**Background:** Tumor budding is considered a prognostic factor in several solid cancer types. However, we lack comprehensive information on the importance of tumor budding in cholangiocarcinoma. Therefore, we aimed to assess the prognostic value of tumor budding in intrahepatic and extrahepatic cholangiocarcinomas and to evaluate its correlations with other clinicopathological parameters.

**Methods:** We monitored 219 patients who underwent surgery for intrahepatic or extrahepatic cholangiocarcinoma at the Pusan National University Hospital between 2012 and 2021. Tumor budding was evaluated using the International Tumor Budding Consensus Conference scoring system. Tumor budding was classified into low (0–4), intermediate (5–9), and high (≥10). For statistical analysis, tumor budding was divided into two groups based on the cut-off value of 10 (lower: 0–9 vs. higher: ≥10). The correlations between clinicopathological parameters were examined using the chi-square and Fisher’s exact test. The prognostic values of the variables were analyzed using the log-rank test and Cox regression analysis.

**Results:** Low, intermediate, and high tumor buddings were identified in 135 (61.6%), 63 (28.8), and 21 (9.6%), patients, respectively. Higher tumor budding was related to the presence of lymphatic invasion (*p* = 0.017), higher tumor grade (*p* = 0.001), higher N category (*p* = 0.034). In the univariable and multivariable analyses, higher tumor budding was associated with shorter disease-free survival in 97 (44.3%) patients who underwent R0 resection (*p* < 0.001 and *p* = 0.011). Tumor budding did not significantly correlate with disease-specific survival in entire patients.

**Conclusion:** Tumor budding may serve as a prognostic factor for intrahepatic and extrahepatic cholangiocarcinomas treated with R0 resection.

## Introduction

Cholangiocarcinoma (CC) is a malignant neoplasm originating from epithelial cells lining the biliary tract. CC is divided into intrahepatic CC (iCC) and extrahepatic CC (eCC) depending on its anatomical location. Over the past few decades, the overall incidence of CC, particularly iCC, has gradually increased worldwide [1]. CC is associated with a poor prognosis, with a 5-year survival rate of 10%–49% for iCC and 20%–40% for eCC [2]. However, the prognostic factors for CC remained poorly understood.

Tumor budding (TB) is defined as a single tumor cell or a cluster of fewer than 5 cells that are observed separately from the tumor mass exhibiting invasion [3]. TB is a histopathological phenomenon of the epithelial-mesenchymal transition (EMT), the process by which epithelial cells are transformed into mesenchymal stem cells. During this process, the cells tend to migrate and invade instead of losing cell polarity and intercellular adhesion. EMT is a crucial developmental process; however, in the case of solid tumors, it promotes tumor growth by increasing invasive and metastatic activity. Since Imai et al. [4] mentioned it first, TB has been actively investigated mainly in patient with colorectal cancer. TB is associated with lymph node metastasis, distant metastasis, local recurrence, and poor prognosis regardless of the TNM stage in patients with colorectal cancer [5]. In 2016, a consensus was reached to objectively and accurately measure TB in patients with colorectal cancer [6]. By introducing this concept, studies on the prognostic significance of TB in various cancer types, such as pancreatic, gallbladder, esophageal, and gastric cancers, have been conducted [7–10]. Although relevant studies have recently been performed to evaluate the importance of TB in CC, the number remains limited, and only 10 papers have been published to date (as of March 2023) [11–19].

Hence, we aimed to investigate the relationship between TB and clinicopathological parameters and the prognostic significance of TB in iCC and eCCs.

## Materials and methods

### Case selection

Based on the medical records, we selected patients who underwent surgery and histopathological examination for biliary tract cancer at Pusan National University Hospital from January 2012 to December 2021 through the medical records. Patients diagnosed with cholangiocarcinoma arising in the intrahepatic, perihilar, and distal extrahepatic bile ducts were included in this study. Patients diagnosed with gallbladder or cystic duct cancer were excluded. Patients who received neoadjuvant chemotherapy; were diagnosed with cancers, such as undifferentiated, squamous cell, or neuroendocrine carcinomas other than adenocarcinomas, and with missing data were excluded.

### Clinicopathological parameters

Data on age, sex, date of local recurrence, distant metastasis, date of last hospital visit or death, and cause of death were obtained. One pathologist (J.H.Ahn) reviewed all available hematoxylin and eosin (H&E) slides with pathologic reports to determine the tumor location, size, histologic grade, lymphatic invasion status, venous invasion status, perineural invasion status, and resection marginal status. Depending on the tumor location, each patient was classified into the iCC group or eCC group. The eCC group included the CC arising from the perihilar and distal extrahepatic bile ducts. Pathologic TNM categories and overall stages were reassessed according to the eighth edition of the American Joint Committee on Cancer [20].

### Evaluation of tumor budding

TB was basically evaluated according to the criteria recommended by the 2016 International Tumor Budding Consensus (ITBCC) [6]. The H&E-stained slide with the highest degree of budding at the invasive front was selected. Hotspots were identified at low magnification, and the number of TB was counted at ×200 magnification. Patients with TB were classified into the low (0–4), intermediate [5–9], and high (≥10) groups. For statistical analysis, TB was reclassified into lower (0–9) and higher (≥10) groups. Two pathologists (K. B. Kim and J. H. Ahn) independently evaluated all available slides containing the tumor samples of each patient. However, they were blinded to all clinical data and the assessment results of the other pathologist. The concordance rate was 89.4%. In cases of disagreement, the two pathologists discussed and reached a consensus.

### Statistical analysis

The correlations between TB and other parameters were evaluated using the chi-square and Fisher’s exact test. Kaplan-Meier analysis was performed to compare the differences in disease-free survival (DFS) and disease-specific survival (DSS) between the TB groups. Univariable Cox regression analysis was performed to identify the parameters that could affect the DFS and DSS. Multivariable Cox regression analysis was performed using parameters considered significant result in the univariable analysis. All statistical analyses were performed using IBM SPSS Statistics (version 27.0; IBM Corp., New York, United States). A *p*-value <0.05 was considered significant.

### Research ethics

This study was approved by the Institutional Review Board of Pusan National University Hospital (approval number 2302-001-123) and performed in accordance with the Declaration of Helsinki.

## Results

### Clinicopathologic characteristics

Table 1 shows the clinicopathologic characteristics of the entire cohort of this study. This study included 219 patients with a mean age was 74.3 years (range: 45 and 96 years). Of the total patients, 137 (62.6%) were male and 82 (37.4%) were female. A total of 49 (22.4%) patients had iCC, while 170 (77.6%) had eCC. Forty-four (20.1%), 138 (63.0%), and 37 (16.9%) patients had well, moderately, and poorly differentiated adenocarcinomas, respectively. Lymphatic, venous, and perineural invasions were identified in 81 (37.9%), 103 (47.0%), and 172 (78.5%) patients, respectively. A total of 97 (44.3%) patients underwent R0 resection. A total of 66 (30.1%) patients had the T1 category, 93 (42.5%) with the T2 category, 57 (26.0%) with the T3 category, and 3 (1.4%) with the T4 category. N0, N1, and N2 categories were identified in 147 (67.1%), 53 (24.2%), and 19 (8.7%) patients, respectively. According to the AJCC staging system, 53 (24.2%) patients had stage I CC, 121 (55.3%) had stage II CC, 40 (18.3%) had stage III CC, and 5 (2.3%) had stage IV CC.

**TABLE 1:** Clinicopathological characteristics.

Parameter	N = 219	%
Age (mean; range) (yr)	74.3; 45–96	
< 60	17	7.8
≧ 60	202	92.2
Sex		
Male	137	62.6
Female	82	37.4
Location		
Intrahepatic	49	22.4
Extrahepatic	170	77.6
Size (mean; range) (mm)	37.21; 3–152	
<50	174	79.5
≧50	45	20.5
Grade		
Well differentiated	44	20.1
Moderately differentiated	138	63.0
Poorly differentiated	37	16.9
Lymphatic invasion		
No	136	62.1
Yes	81	37.9
Venous invasion		
No	116	53.0
Yes	103	47.0
Perineural invasion		
No	47	21.5
Yes	172	78.5
Resection marginal status		
Negative (R0)	97	44.3
Positive (R1 or R2)	122	55.7
Tumor budding		
Low (0–4)	135	61.6
Intermediate (5–9)	63	28.8
High (≥10)	21	9.6
T category		
1	66	30.1
2	93	42.5
3	57	26.0
4	3	1.4
N category		
0	147	67.1
1	53	24.2
2	19	8.7
Overall stage		
I	53	24.2
II	121	55.3
III	40	18.3
IV	5	2.3

### Tumor budding

**FIGURE 1:**
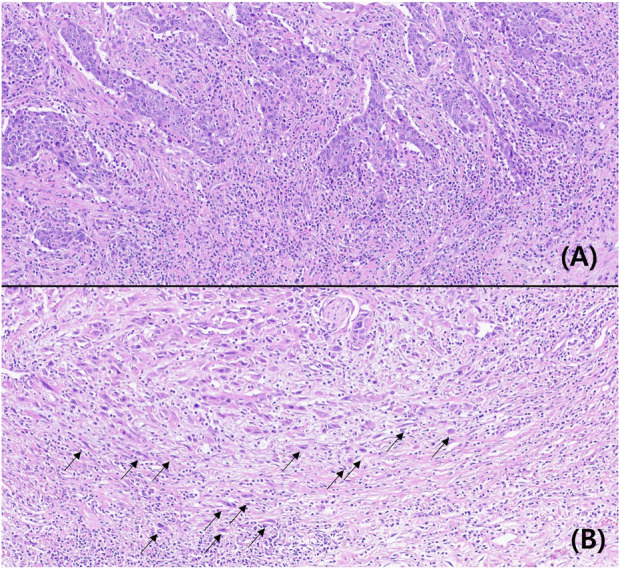
Microscopic findings of low **(A)** and high **(B)** tumor buddings (Hematoxylin-eosin, ×200, arrow: tumor budding).

TB was not observed in 41 patients. At least one tumor bud was observed in 178 patients. Based on the ITBCC scoring system, 135 (61.6%), 63 (28.8%), and 21 (9.6%) patients had low, intermediate, and high TB, respectively. According to the TB classification for statistical analysis, the lower and higher TB groups were 198 (90.4%) and 21 (9.6%) patients. The typical findings are shown in Figure 1.

### Association between tumor budding and other parameters

The correlations between TB and clinicopathological parameters are presented in Table 2. Higher TB was associated with higher tumor grade (*p* = 0.001), lymphatic invasion (*p* = 0.017), and higher N category (*p* = 0.034). In patients who had venous and perineural invasion, a higher TB tended to be more prevalent, but the difference was not significant (*p* = 0.058 and 0.050, respectively). Age, sex, tumor location, size, resection marginal status, T category, and the overall stage had no correlation with TB.

**TABLE 2:** Association between tumor budding and clinicopathological parameters.

Parameter	Lower TB^a^ (%) (*n* = 198, 90.4%)	Higher TB (%) (*n* = 21, 9.6%)	*p*-value
Age (mean; range) (yr)			
<60	15 (88.2)	2 (11.8)	*0.670*
≧60	183 (90.6)	19 (9.4)	
Sex			*0.095*
Male	120 (87.6)	17 (12.4)	
Female	78 (95.1)	4 (4.9)	
Location			*0.423*
Intrahepatic	46 (93.9)	3 (6.1)	
Extrahepatic	152 (89.4)	18 (10.6)	
Size (mean; range) (mm)			0.228
<50	155 (91.7)	14 (8.3)	
≧50	43 (86.0)	7 (14.0)	
Grade			0.001*
Well differentiated	44 (100.0)	0 (0.0)	
Moderately differentiated	126 (91.3)	12 (8.7)	
Poorly differentiated	28 (75.7)	9 (24.3)	
Lymphatic invasion			0.017*
No	128 (94.1)	8 (5.9)	
Yes	70 (84.3)	13 (15.7)	
Venous invasion			*0.058*
No	109 (94.0)	7 (6.0)	
Yes	89 (86.4)	14 (13.6)	
Perineural invasion			*0.052*
No	46 (97.9)	1 (2.1)	
Yes	152 (88.4)	20 (11.6)	
Resection marginal status			*0.127*
Negative (R0)	91 (93.8)	6 (6.2)	
Positive (R1 or R2)	107 (87.7)	15 (12.3)	
T category			*0.505*
1	62 (93.9)	4 (6.1)	
2	84 (90.3)	9 (9.7)	
3	49 (86.0)	8 (14.0)	
4	3 (100.0)	0 (0.0)	
N category			0.034*
0	138 (93.9)	9 (6.1)	
1	45 (84.9)	8 (15.1)	
2	15 (78.9)	4 (21.1)	
Overall stage			*0.130*
I	50 (94.3)	3 (5.7)	
II	108 (89.3)	13 (10.7)	
III	37 (92.5)	3 (7.5)	
IV	3 (60.0)	2 (40.0)	

^a^ Tumor budding.

### Survival according to tumor budding

**FIGURE 2:**
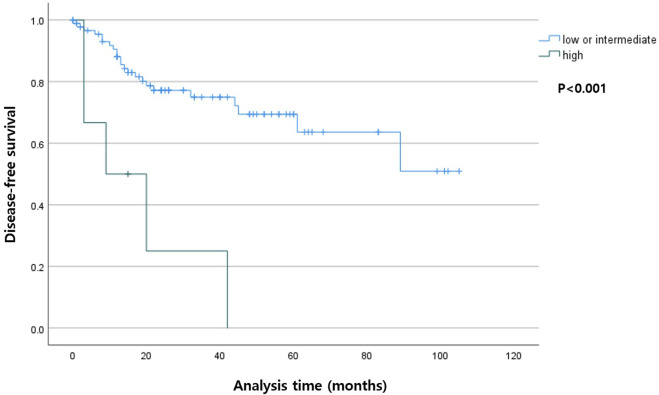
Kaplan-Meier curve depicting disease-free survival according to the tumor budding status in patients with R0 resection.

**FIGURE 3:**
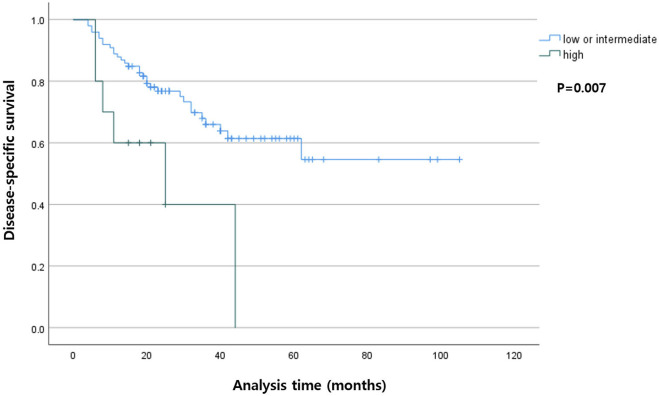
Kaplan-Meier curve showing disease-specific survival according to the tumor budding status in the entire cohort.

Among the patients with R0 resection, the higher TB group had shorter DSS (*p* < 0.001) compared with that in the lower TB groups. In the entire cohort, the higher TB patients had shorter DFS (*p* = 0.007). The Kaplan-Meier curves for DSS and DFS are presented in Figures 2, 3, respectively.

### Factors associated with disease-free survival

Table 3 shows the association between the clinicopathological parameters and DFS in 97 patients who underwent R0 resection. In the univariable analysis, poor differentiation, venous invasion, TB, advanced [3, 4] T category, and N category were associated with DFS. In multivariable analysis, the presence of venous invasion, and higher TB and N category were related to shorter DFS.

**TABLE 3:** Factors associated with disease-free survival in patients with R0 resection.

	Univariable analysis			Multivariable analysis			
	HR^a^	95% CI^b^	*p*-value	HR	95% CI		*p-*value
Age (yr)							
(<60/≧60)	1.148	0.271–4.861	*0.851*				
Sex							
(male/female)	0.977	0.428–2.230	*0.955*				
Location							
(intra-/extra-hepatic)	1.601	0.679–3.775	*0.282*				
Size							
(<50/≧50)	2.048	0.861–4.871	*0.098*				
Grade							
Well differentiated	Reference			Reference			
Moderately differentiated	1.547	0.522–4.588	*0.431*	0.723	0.217–2.412		*0.598*
Poorly differentiated	3.993	1.099–14.508	0.035*	1.802	0.420–7.727		*0.428*
Lymphatic invasion							
(no/yes)	1.927	0.881–4.213	*0.095*				
Venous invasion							
(no/yes)	3.989	1.829–8.700	<0.001*	2.887	1.259–6.621		0.012*
Perineural invasion							
(no/yes)	1.302	0.587–2.884	*0.515*				
Tumor budding							
(lower/higher)	5.755	2.133–15.529	<0.001*	3.969	1.375–11.455		0.011*
T category							
1	Reference			Reference			
2	1.936	0.798–4.698	*0.144*	1.229	0.463–3.261		*0.679*
3–4	2.712	1.018–7.228	0.046*	1.076	0.301–3.843		*0.910*
N category							
0	Reference			Reference			
1	3.379	1.387–8.237	0.007*	2.635	1.045–6.647		0.040*
2	4.040	1.158–14.090	0.028*	4.440	1.234–15.970		0.022*
Overall stage							
I	Reference						
II	2.174	0.927–5.101	*0.074*				
III–IV	3.116	0.795–12.220	*0.103*				

^a^ Hazard ratio.

^b^ Confidence interval.

### Factors associated with disease-specific survival

Table 4 presents the association between the clinicopathological parameters and DSS in all 219 patients. In the univariable analysis, lymphatic, venous, and perineural invasion status; resection marginal status; TB; and T and N categories and overall stages were correlated with DSS**.** In the multivariable analysis, positive resection marginal status and higher T category were associated with shorter DSS. However, TB was not correlated with DSS in the multivariable analysis.

**TABLE 4:** Factors associated with disease-specific survival.

	Univariable analysis			Multivariable analysis		
	HR^a^	95% CI^b^	*p-*value	HR	95% CI	*p-*value
Age (yr)						
(<60/≧60)	0.538	0.223–1.300	*0.162*			
Sex						
(male/female)	0.892	0.462–1.724	*0.734*			
Location						
(intra-/extra-hepatic)	1.107	0.486–2.525	*0.808*			
Size						
(<50/≧50)	1.201	0.592–2.437	*0.611*			
Grade						
Well differentiated	Reference					
Moderately differentiated	1.108	0.490–2.509	*0.805*			
Poorly differentiated	2.249	0.835–6.060	*0.109*			
Lymphatic invasion						
(no/yes)	2.493	1.287–4.829	0.005*	1.175	0.423–3.266	*0.757*
Venous invasion						
(no/yes)	4.246	2.032–8.875	<0.001*	1.947	0.820–4.624	*0.131*
Perineural invasion						
(no/yes)	5.115	1.566–16.707	0.003*	0.713	0.169–3.014	*0.645*
Resection marginal status						
(negative/positive)	6.926	2.684–17.857	0.000*	4.893	1.613–15.092	0.002*
Tumor budding						
(lower/higher)	3.134	1.291–7.608	0.008*	1.428	0.494–4.128	*0.511*
T category						
1	Reference			Reference		
2	3.126	1.044–9.356	0.042*	3.374	1.061–10.737	0.039*
3–4	7.229	2.414–21.652	<0.001*	5.262	1.618–17.112	0.006*
N category						
0	Reference			Reference		
1	3.462	1.668–7.187	0.001*	1.670	0.733–3.807	*0.222*
2	9.629	3.750–24.725	<0.001*	6.667	2.284–19.461	0.001*
Overall stage						
I	Reference			Reference		
II	9.076	1.217–67.697	0.031*	2.262	0.159–32.243	*0.547*
III–IV	28.414	3.659–218.591	0.001*	2.740	0.173–43.473	*0.475*

^a^ Hazard ratio.

^b^ Confidence interval.

## Discussion

Our findings suggested that a higher TB was related to factors indicating aggressiveness, such as lymphatic invasion, higher tumor grade, and higher N category. In patients who underwent R0 resection, the higher TB group showed a shorter DFS compared with that of the lower TB group.

TB is an EMT phenotype. Anchorage-dependent cells undergo a programmed cell death process, called anoikis, when separated from the surrounding extracellular matrix. EMT provides the characteristics of stem cells, while destroying the intercellular junctions and avoiding anoikis in the tumor microenvironment, thus resulting in the acquisition of mobility and invasive phenotypes. TB cells in colorectal and gastric cancers overexpress tyrosine kinase receptor B (TrkB), an indicator of anoikis resistance, which allows TB cells to survive [21, 22]. Surviving TB cells are inevitably associated with invasiveness. Microscopically, TB cells are often observed near the lymphatic or blood vessels, which suggests the possibility of intravasation and spreading to distant tissues [22]. Lugli et al. suggested that TB is a good indicator of aggressiveness in rectal cancer, demonstrating that it is associated with the number of involved nodes, extramural spread, lymphocytic infiltration, and tumor differentiation [3]. Okudo et al. reported that TB occurs more frequently observed in the cases of poor differentiation at the invasive front of biliary tract cancer [16]. Agostini-Vulaj et al. also reported that TB is associated with high tumor grade, lymphovascular invasion, and perineural invasion in iCC and eCCs [11]. In this study, TB was significantly associated with lymphatic invasion, and higher tumor grade and N category. Higher TB was associated with the presence of venous and perineural invasion but did not show statistical significance in this study.

Okudo et al. are the first team to evaluate the prognostic impact of TB in the CC [16]. They demonstrated that the ≧5 TB foci were as an independent predictor in overall survival. Ogino et al. also reported that patients with perihilar and distal duct CC who had a high TB grade had a short postoperative overall survival [15]. Ito et al. reported that high TB was correlated with poor DSS in patients with perihilar CC; moreover, the DSS rate in the high TB group was similar to that of the group without resection [12]. In this study, DSS was not correlated with TB in the entire cohort. However, the DFS was significantly associated with TB in patients who underwent R0 resection.

All studies investigating TB in patients with CC published thus far graded the TB based on ITBCC scoring system [11–15, 17–19, 23]. Most of these studies classified TB into low and intermediate/high groups based on the cut-off value of 5 [11, 12, 14, 15, 18]. Conversely, Kosaka et al. reported a difference in the survival outcomes between low/intermediate and high TB groups, using a cut-off value of 10 in their multicenter study [13]. As in Kosaka’s study, we divided patients with TB into low/intermediate and high groups. Findings from previous studies and the present study suggest that the ITBCC method is appropriate for TB cell counting in cholangiocarcinoma. Higher TB levels were correlated with worse survival. However, few studies had revealed the correlation between TB and survival when patients with TB were divided into three groups according to the ITBCC scoring system. Previous studies had demonstrated a good correlation with survival when TB was divided into two groups based on a cut-off value of 5 or 10. Therefore, for CC, further investigation is required to confirm whether the ITBCC scoring system should be applied as it is or whether a revised system should be established.

Recently, Zlobec et al. proposed the revised ITBCC scoring system [24]. They reported that dividing TB into four categories, including “zero-buds,” is more informative on tumor behavior than the previous ITBCC scoring system. This study included 41 (18.7%) patients with zero buds. However, in this study, there was no difference between DFS and DSS in the zero-buds and lower-TB (1–9) groups (Supplementary Material S1).

Ogino et al. reported differences in prognostic stratification with TB in perihilar CC and distal CC [15]. These differences were in term of anatomical location and histological characteristics; for example, the connective tissue surrounding the proximal bile duct is less dense than the distal bile duct. On the contrary, Agostini-Vulaj et al. reported that TB occurred more in eCC than iCC [11]. In this study, there was no difference in the frequency of TB according to the anatomical location. Therefore, one cannot conclude on the difference in the frequency of TB.

This study has several limitations. First, we investigated the eCC and iCC, including large- and small-duct types. CCs may have varying carcinogenic mechanisms depending on their location. Particularly, in iCC, the large duct type is similar to perihilar CC, while the small duct type is similar to the adenocarcinoma component of combined hepatocellular-cholangiocarcinoma. However, considering these differences, the number of cases in each location was insufficient to conduct this study according to the tumor location. Second, patients who had incomplete resection accounted for over half of the cohort in this study. Therefore, the data used for analyzing the DFS were limited. Hence, further investigations are required to address these limitations.

In conclusion, our findings suggested that higher TB levels in patients with CC are associated with aggressiveness and worse DFS. Moreover, TB may have prognostic implications in CC.

## Data Availability

The raw data supporting the conclusion of this article will be made available by the authors, without undue reservation.
