# Supplementary material for: Tumor budding as a predictor of disease-free survival in patients with cholangiocarcinoma
Source: Pathol Oncol Res. 2023 May 18;29:1611216. doi: 10.3389/pore.2023.1611216 (PMC10232744; doi:10.3389/pore.2023.1611216)
Supplement: Supplementary file 1 [file DataSheet1.docx]

Supplementary Material

Tumor budding as a predictor of disease-free survival in patients with cholangiocarcinoma

Kyung Bin Kim^1^, Ji Hyun Ahn^1*^, Soon Wook Kwon^1^, Su Ji Lee^1^, Yury Lee^1^, Seo Young Park^1^, Ahrong Kim^1,2^, Kyung Un Choi^1,2^, Chang Hun Lee^1,2^, Gi Yeong Huh^1,2^

^1^Department of Pathology, Pusan National University Hospital, Busan, Republic of Korea

^2^Department of Pathology, Pusan National University School of Medicine, Yangsan-si, Gyeongsangnamdo, Republic of Korea

*** Correspondence:**Ji Hyun Ahn
[jvcjh@hanmail.net](mailto:jvcjh@hanmail.net)

**Supplementary Figure 1.** There was no difference according to tumor buddings in disease-free survival.


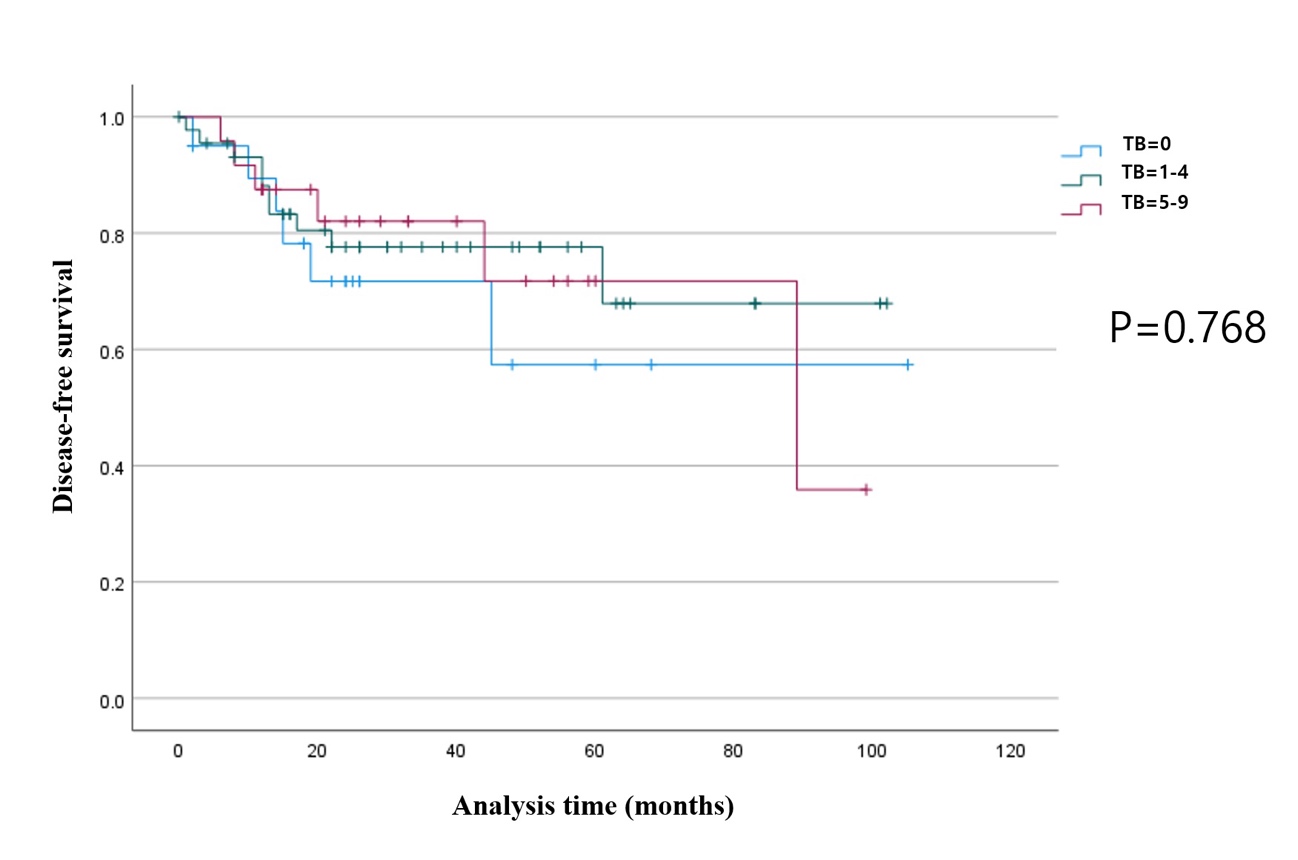


**Supplementary Figure 2.**  There was no difference according to tumor buddings in disease-specific survival.

**
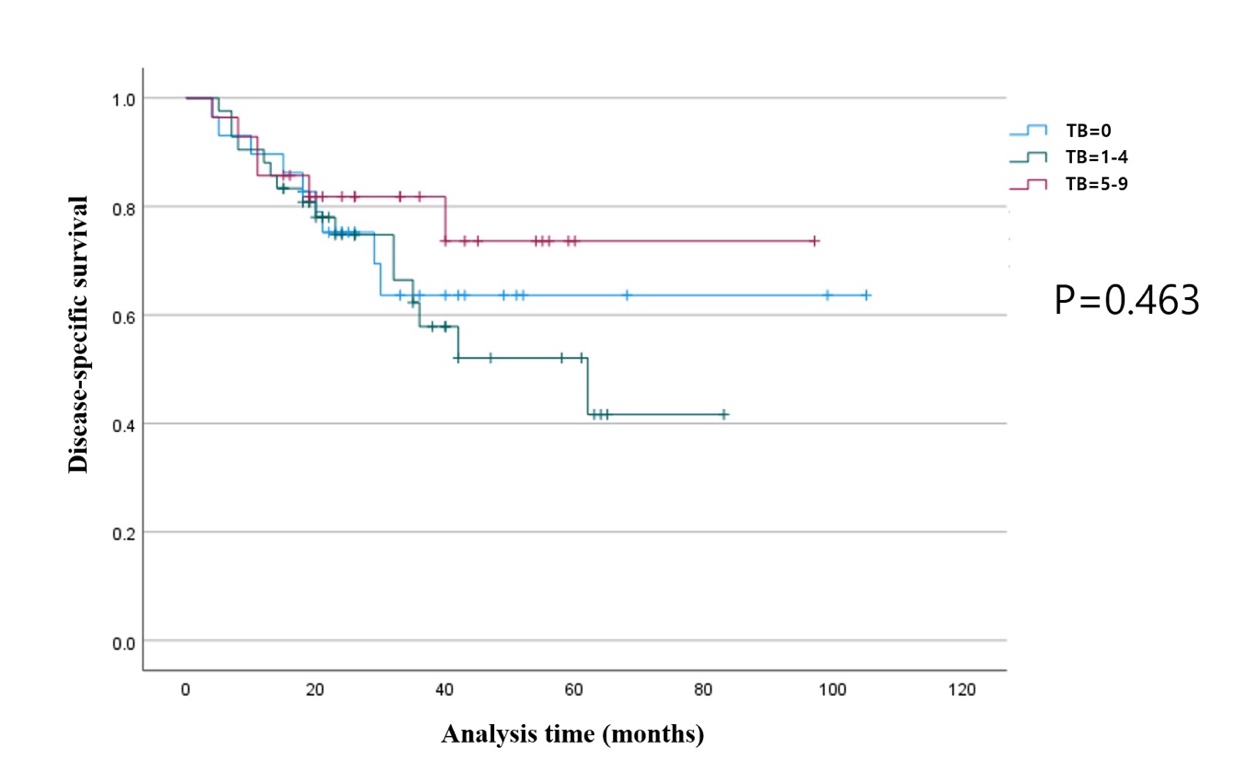
**
